# Intra‐cardiac thrombosis in the setting of heparin‐induced thrombocytopenia

**DOI:** 10.1002/ccr3.4677

**Published:** 2021-08-23

**Authors:** Amr Mohamed

**Affiliations:** ^1^ Department of Internal Medicine Rochester General Hospital Rochester NY USA

**Keywords:** atrial fibrillation, heparin, thrombocytopenia, thrombosis

## Abstract

Key clinical message is to know that HIT is a massive thrombotic storm. Atrial fibrillation cardioversion might not be safe in HIT using the standard 48‐hour cutoff from arrhythmia onset. Also, the case serves as a reminder of how to suspect, diagnose, and treat HIT.

## Graphical abstract

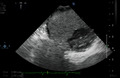

## 1 INTRODUCTION

A 56‐year‐old man had been admitted to the hospital with heart failure exacerbation. He had been on telemetry since admission and had been in sinus rhythm. He had a loop recorder for the last three months with no evidence of atrial fibrillation. He had acute onset atrial fibrillation in the hospital, off‐note he had been on enoxaparin for thromboprophylaxis since admission. He complained of calf pain on day 8, and a duplex lower extremities showed right common femoral deep venous thrombosis. Laboratory work is significant for a platelet count drop from 400 on day 4 to 130 on day 8 of hospital stay. The rest of the laboratory work was unremarkable.

**FIGURE 1:**
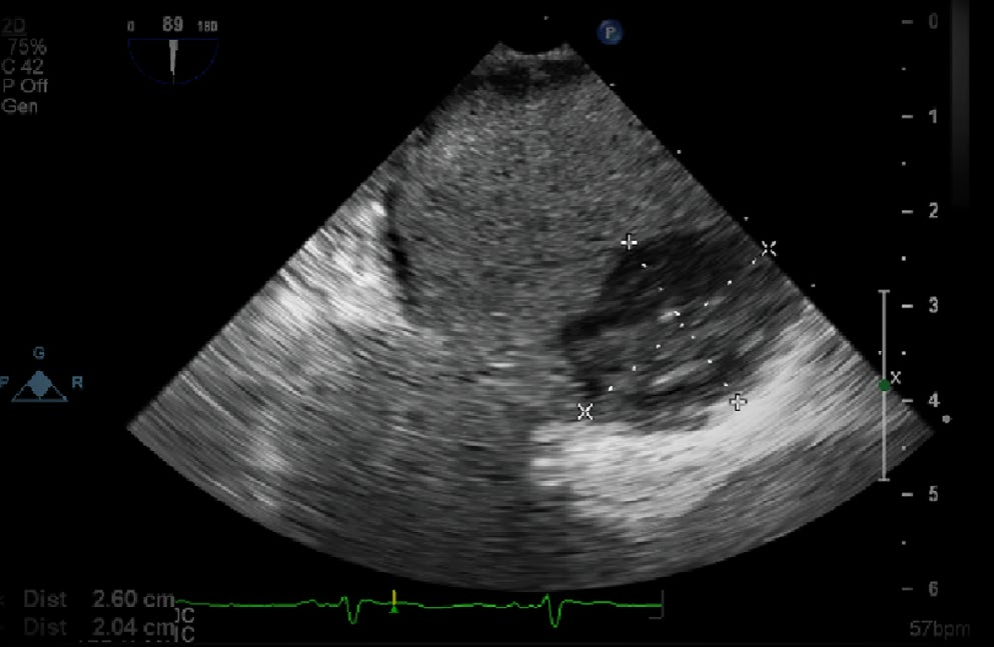
is showing a transoesophageal echocardiogram with evidence of huge left atrial thrombus

Despite the concise duration of atrial fibrillation, we decided against cardioversion without a transoesophageal echo(TEE) as we were suspecting heparin‐induced thrombocytopenia(HIT), and we were afraid of thrombotic complications. TEE showed a massive left atrial thrombus, as shown in Figure 1.

His 4T score was eight, which is a high probability, and platelet factor 4 ELISA IgG optical density came back positive at 2.2, which confirms a diagnosis of HIT. He was started on bivalirudin and was later shifted to apixaban for chronic atrial fibrillation anticoagulation.

The key clinical message is to know that HIT is a massive thrombotic storm.^1^ Atrial fibrillation cardioversion might not be safe in HIT using the standard 48‐hour cutoff from arrhythmia onset. Also, the case serves as a reminder of how to suspect, diagnose, and treat HIT.

## CONFLICT OF INTEREST

None.

## ETHICAL STATEMENT

Patient verbal consent had been obtained to use the material.

## AUTHOR CONTRIBUTIONS

The author had been responsible for data collection, analysis, and presentation.

## Data Availability

The data that support the findings of this study are available from the corresponding author upon reasonable request.
